# Exploring the Phenotypic and Genetic Variabilities in Yield and Yield-Related Traits of the Diallel-Crossed F_5_ Population of Aus Rice

**DOI:** 10.3390/plants12203601

**Published:** 2023-10-17

**Authors:** Md. Arifur Rahman Khan, Apple Mahmud, Uttam Kumar Ghosh, Md. Saddam Hossain, Md. Nurealam Siddiqui, A. K. M. Aminul Islam, Touhidur Rahman Anik, Md. Mezanur Rahman, Anket Sharma, Mostafa Abdelrahman, Chien Van Ha, Mohammad Golam Mostofa, Lam-Son Phan Tran

**Affiliations:** 1 Department of Agronomy, Bangabandhu Sheikh Mujibur Rahman Agricultural University, Gazipur 1706, Bangladesh; applemahmud885@gmail.com (A.M.); uttam@bsmrau.edu.bd (U.K.G.); saddam.agr@bsmrau.edu.bd (M.S.H.); 2 Institute of Genomics for Crop Abiotic Stress Tolerance, Department of Plant and Soil Science, Texas Tech University, Lubbock, TX 79409, USA; tanik@ttu.edu (T.R.A.); mdmerahm@ttu.edu (M.M.R.); mosabdel@ttu.edu (M.A.); chien.ha@ttu.edu (C.V.H.); 3 Department of Biochemistry and Molecular Biology, Bangabandhu Sheikh Mujibur Rahman Agricultural University, Gazipur 1706, Bangladesh; nuralambmb@bsmrau.edu.bd; 4 Department of Genetics and Plant Breeding, Bangabandhu Sheikh Mujibur Rahman Agricultural University, Gazipur 1706, Bangladesh; aminulgpb@bsmrau.edu.bd; 5 MSU-DOE Plant Research Laboratory, Michigan State University, East Lansing, MI 48824, USA; 6 Department of Biochemistry and Molecular Biology, Michigan State University, East Lansing, MI 48824, USA

**Keywords:** Aus rice, genotypic coefficient of variation, grain yield, phenotypic coefficient of variation, rainfed

## Abstract

Rice (*Oryza sativa*) is a major crop and a main food for a major part of the global population. Rice species have derived from divergent agro-climatic regions, and thus, the local germplasm has a large genetic diversity. This study investigated the relationship between phenotypic and genetic variabilities of yield and yield-associated traits in Aus rice to identify short-duration, high-yielding genotypes. Targeting this issue, a field experiment was carried out to evaluate the performance of 51 Aus rice genotypes, including 50 accessions in F_5_ generation and one short-duration check variety BINAdhan-19. The genotypes exhibited a large and significant variation in yield and its associated traits, as evidenced by a wide range of their coefficient of variance. The investigated traits, including days to maturity (DM), plant height (PH), panicle length (PL) and 1000-grain weight (TW) exhibited a greater genotypic coefficient of variation than the environmental coefficient of variation. In addition, the high broad-sense heritability of DM, PH, PL and TW traits suggests that the genetic factors significantly influence the observed variations in these traits among the F_5_ Aus rice accessions. This study also revealed that the grain yield per hill (GY) displayed a significant positive correlation with PL, number of filled grains per panicle (FG) and TW at both genotype and phenotype levels. According to the hierarchical and K-means cluster analyses, the accessions BU-R-ACC-02, BU-R-ACC-08 and R2-36-3-1-1 have shorter DM and relatively higher GY than other Aus rice accessions. These three accessions could be employed in the ongoing and future breeding programs for the improvement of short-duration and high-yielding rice cultivars.

## 1. Introduction

The cereal crop rice (*Oryza sativa*) is a member of the *Poaceae* family. A larger part of the people in the world, mainly in Asian countries, consume rice as a staple food, making it the third most popular cereal grain crop worldwide [1]. Rice provides food, income and employment to 200 million families in underdeveloped nations [2]. Rice is an important cereal crop in Bangladesh, and the country ranks third in the world in terms of the amount of rice production [3]. In Bangladesh, 75% of the entire crop land, around 11 million hectares, is utilized for rice cultivation [4]. It is also estimated that rice production occupies more than 80% of the country’s irrigated area [5]. Three rice-growing seasons, namely Aus, Aman and Boro, are adopted in Bangladesh to cultivate rice all year long (rice types are named Aus, Aman and Boro according to their growing seasons). Among the three rice types, Aus is accountable for 2.60 million tons of production and covers an area of 1.18 million hectares, with an average yield of 2.20 tons per hectare for the 2019–2020 crop year [6]. The population of Bangladesh is expanding by two million every year [7]. Under this circumstance, rice production must increase to meet the rising demand of the population. Boro is the main type of rice in Bangladesh, in which cultivation puts much pressure on groundwater-based irrigation, as Boro-type rice relies entirely on irrigation for production. To alleviate groundwater pressure, Boro-cultivating fields should be converted into Aus-cultivating ones to ensure the country’s food supply [8]. However, the productivity of Aus rice has been falling for several years since Aus rice is cultivated only in highland areas during the summer under rainfed conditions. In addition, much of the summer-time rice planted in the Aus season has a low yield and a protracted maturation period [9]. Thus, finding Aus genotype(s) with a short-duration and high-yielding potential is essential to increase sustainable rice production in Bangladesh.

Aus rice breeding is difficult due to the lack of knowledge about various environmental conditions that influence upland rice cultivation. Yield is a complicated feature, as various morphological, physiological and agronomic attributes impact rice’s ability to maintain productivity, particularly under various environmental conditions [10]. Indeed, a thorough investigation of genetic makeup, genetic diversity and the interplay of agronomic factors influencing yield among the already-grown cultivars is necessary to develop high-yielding rice varieties under these circumstances [11]. Rice breeders usually prefer morphological traits-based genetic diversity analyses because these methods are quick, easy to analyze and economical [12]. Numerous techniques can be used to analyze the genetic diversity of breeding lines, such as multivariate analysis that simultaneously looks at all random components [13]. To reduce variations between groups, cluster analysis is a technique for identifying and classifying variables based on how similar their properties are. Cluster analysis also delivers on how genotypes are interrelated [14]. It is also advantageous to select rice genotypes with admirable agronomic characteristics. Additionally, the multivariate analysis is used to uncover promising features as selection criteria to boost agricultural yields [15,16].

Assessing phenotypic and genetic diversities and estimating the relationship between cultivar-specific yields and their constituent parts are particularly important for developing new crop genotypes [17]. In the current study, we investigated and evaluated the phenotypic and genetic variabilities of yield and yield-related traits of 51 genotypes, including 50 Aus rice accessions in F_5_ generation and one check variety BINAdhan-19, considering the benefits of multivariate analyses to identify short-duration and high-yielding genotypes.

## 2. Results

### 2.1. Phenotypic Performance of the F_5_ Aus Rice Population

In the present investigation, a comprehensive analysis was conducted on 10 agronomical traits, including days to maturity (DM), plant height (PH), total tillers per hill (TT), effective tillers per hill (ET), panicle length (PL), number of filled grains per panicle (FG), number of unfilled grains per panicle (UFG), 1000-grain weight (TW), grain yield per hill (GY) and straw yield per hill (SY) (Table 1). This extensive study was performed across 50 F_5_ Aus rice accessions and one standard short-duration check variety, BINAdhan-19. All accessions were cultivated under field conditions to discern them in terms of yield potential and growth duration. One-way analysis of variance (ANOVA) utilizing the mean sum of squares revealed significant differences in the 10 investigated agronomical traits across the various F_5_ Aus rice genotypes (Supplementary Table S1). This result indicated a wide variability among the rice genotypes, suggesting that this population is suitable for further germplasm selection. Because the one-way ANOVA did not allow us to differentiate the rice genotypes from each other, we conducted a Fisher’s least significant difference (LSD_0.05_) test to perform pairwise comparisons between the genotypes (Table 1). All the 50 F_5_ rice genotypes exhibited significant variations in DM and PH traits compared with the standard short-duration and semi-dwarf BINAdhan-19 check variety, which were evidenced by the mean difference (MD) values exceeding the Fisher’s LSD_0.05_ thresholds (Table 1). On the other hand, only a few rice accessions displayed significant variations in ET, FG, GY, PL, SY, TT, TW and UFG traits when compared with the standard BINAdhan-19 check variety, as indicated by their higher MD values than the Fisher’s LSD_0.05_ thresholds (Table 1). Among all investigated traits, the DM showed slight variability within the assessed 51 rice genotypes as indicated by the lowest coefficient of variation (CV), whereas the UFG exhibited pronounced variability as indicated by the highest CV (Table 1). Consistently, across the 51 rice genotypes, the maximum and minimum values observed for DM were 116.00 and 96.00, respectively, implying a narrow range of variation (Supplementary Table S1). In contrast, the maximum and minimum values observed for UFG were 115.00 and 3.00, respectively, indicating a wide range of variation (Supplementary Table S1).

### 2.2. Effects of Genetic and Environmental Components on the Agronomical Traits of Aus Rice Population

The observed variability in the 10 investigated agronomic traits across 51 Aus rice populations may be attributed to genetic and/or non-genetic components. These genetic and non-genetic components can be explained by using a quantitative genetic model, where the phenotypic variance (*σ*^2^*p*) of the Aus rice population is a result of environmental variance (*σ*^2^*e*), genotypic variance (*σ*^2^*g*) and their possible interactions [18]. In this study, the *σ*^2^*g* values of DM, PH, TW and PL traits were higher than their corresponding *σ*^2^*e* values, implying that the discerned diversity in these traits within the Aus rice population predominantly emanated from the genetic effects rather than environmental ones (Table 2). Consistently, the genotypic coefficient of variation (GCV) percentages of DM, PH, PL and TW traits were also higher than their corresponding environmental coefficient of variation (ECV) percentage (Table 2). Broad-sense heritability (*H*^2^) captures the proportion of *σ*^2^*p* due to genetic effects and is expressed as the ratio of *σ*^2^*g* to *σ*^2^*p* [19]. In this study, *H*^2^ exhibited a range of 65.37 to 96.31% in DM, PH, TW and PL traits (Table 2), indicating a substantial genetic influence contributing to the observed diversity within the Aus rice population. Therefore, these traits present a significant potential for distinguishing the genetic differences among different accessions of Aus rice, rendering them valuable indicators for germplasm selection. On the other hand, minimal disparities were evident between the *σ*^2^*g* and *σ*^2^*e* components across ET, SY, TT, GY, FG and UFG traits, suggesting the presence of both genetic and environmental influences on these traits. Likewise, marginal variations were observed in the percentages of ECV and GCV, indicating the joint impact of genetic and environmental factors (Table 2). These findings were consistent with the moderate range of *H*^2^, which spanned from 39.34 to 58.63%, for ET, SY, TT, GY, FG and UFG traits (Table 2).

### 2.3. Phenotype- and Genotype-Based Correlations in the Investigated Agronomical Traits of Aus Rice Population

**Figure 1:**
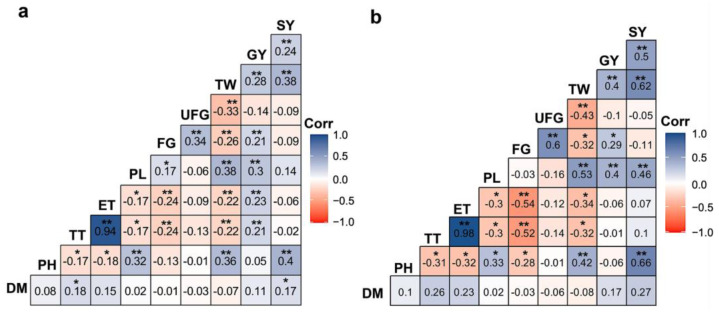
Pearson’s correlation coefficients among the investigated agronomical traits of the 50 F_5_ Aus rice accessions and BINAdhan-19 check variety: (**a**) Phenotype-based correlation and (**b**) genotype-based correlation analyses of 10 agronomical traits across 51 rice genotypes. Color bars denote positive (blue) and negative (red) correlations (Corr). Values represent the Pearson’s correlation coefficients ranging from 1 to −1. Asterisks designate significance levels where * *p* ≤ 0.05 and ** *p* ≤ 0.01, using the ‘corrplot’ package in R version 3.2.0. DM, days to maturity; ET, number of effective tillers per hill; FG, number of filled grains per panicle; GY, grain yield per hill; PH, plant height; PL, panicle length; SY, straw yield per hill; TT, number of total tillers per hill; TW, 1000-grain weight; UFG, number of unfilled grains per panicle.

Results of the phenotype- and genotype-based correlation analyses of 51 Aus rice genotypes using 10 examined agronomical traits are shown in Figure 1a,b. Phenotype-based correlation analysis revealed positive relationships between DM and TT (*r* = 0.18) and DM and SY (*r* = 0.17), implying that the increase in DM might contribute to increasing tiller numbers and, subsequently, SY (Figure 1a). With respect to yield, PL, TW, SY, ET, TT and FG displayed positive relationships with GY (*r* = 0.30, 0.28, 0.24, 0.23, 0.21 and 0.21, respectively) (Figure 1a), implying that these traits might contribute to improving yield in Aus rice accessions. Genotype-based correlation analysis revealed no correlations between DM and other agronomical traits (Figure 1b). On the other hand, SY, TW, PL and FG showed positive correlations with GY (*r* = 0.50, 0.40, 0.40 and 0.29, respectively) (Figure 1b). In general, both phenotype-based and genotype-based correlation analyses revealed that GY was positively correlated with FG, PL, SY and TW traits, indicating these traits might contribute to grain yield in the examined Aus rice genotypes.

### 2.4. Trait–Genotype Interactions Using Cluster Analysis

**Figure 2:**
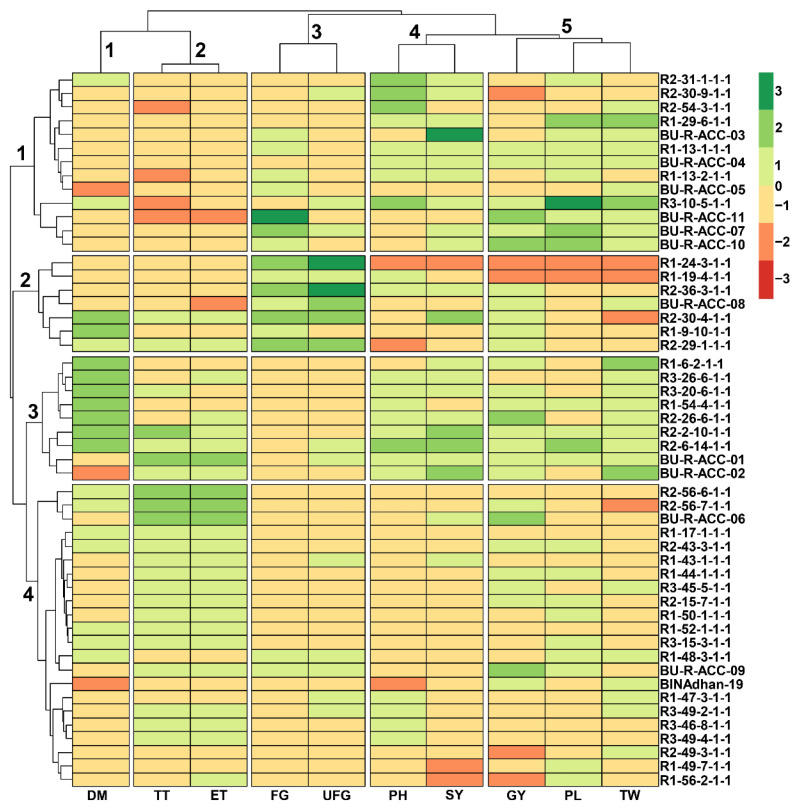
Heatmap hierarchical cluster analysis based on 10 agronomical traits of 50 F_5_ Aus rice accessions and one check variety BINAdhan-19. All 51 genotypes were placed on the Y-axis, whereas all 10 agronomical traits were placed on X-axis. Colors indicate high (green) and low (red) associations between rice genotypes and investigated traits. Numbers on the X-axis and Y-axis represent the number of observed clusters. DM, days to maturity; ET, number of effective tillers per hill; FG, number of filled grains per panicle; GY, grain yield per hill; PH, plant height; PL, panicle length; SY, straw yield per hill; TT, number of total tillers per hill; TW, 1000-grain weight; UFG, number of unfilled grains per panicle.

Heatmap hierarchical clustering using Euclidean distance was carried out to identify the trait–genotype association (Figure 2). All 51 Aus rice genotypes were placed on the Y-axis, whereas all 10 agronomical traits were placed on the X-axis (Figure 2). The 51 genotypes were grouped into two major clusters, and these major clusters were subsequently partitioned into four distinct sub-clusters denoted as 1, 2, 3 and 4 (Figure 2). These sub-clusters accommodated 13, 7, 9 and 22 genotypes, respectively, as illustrated in Figure 2. Similarly, all the agronomical traits were grouped into two major clusters (Figure 2), and these major clusters were subdivided into five sub-clusters labeled 1, 2, 3, 4 and 5 (Figure 2). Specifically, DM was assigned to sub-cluster 1; TT and ET to sub-cluster 2; FG and UFG to sub-cluster 3; PH and SY to sub-cluster 4; and GY, PL and WT were grouped in sub-cluster 5 (Figure 2). In general, most of the rice genotypes in sub-cluster 3 exhibited long DM, whereas most of the genotypes in sub-clusters 1 and 4 exhibited short DM (Figure 2). Among the 51 genotypes, 10 accessions, namely BU-R-ACC-01, BU-R-ACC-02, BU-R-ACC-03, BU-R-ACC-04, BU-R-ACC-05, BU-R-ACC-08, R1-19-4-1-1, R1-29-6-1-1, R2-36-3-1-1 and R3-49-2-1-1, as well as BINAdhan-19 check variety, exhibited shorter DM (Figure 2). With respect to GY, 11 accessions, namely BU-R-ACC-02, BU-R-ACC-06, BU-R-ACC-07, BU-R-ACC-08, BU-R-ACC-09, BU-R-ACC-10, BU-R-ACC-11, R1-13-1-1-1, R1-44-1-1-1, R2-26-6-1-1 and R2-36-3-1-1, produced comparatively higher GY than other accessions (Figure 2). Interestingly, BU-R-ACC-02, BU-R-ACC-08 and R2-36-3-1-1 accessions exhibited both short DM and high GY, implying that these lines are potential for rice breeding programs (Figure 2 and Supplementary Table S2).

**Figure 3:**
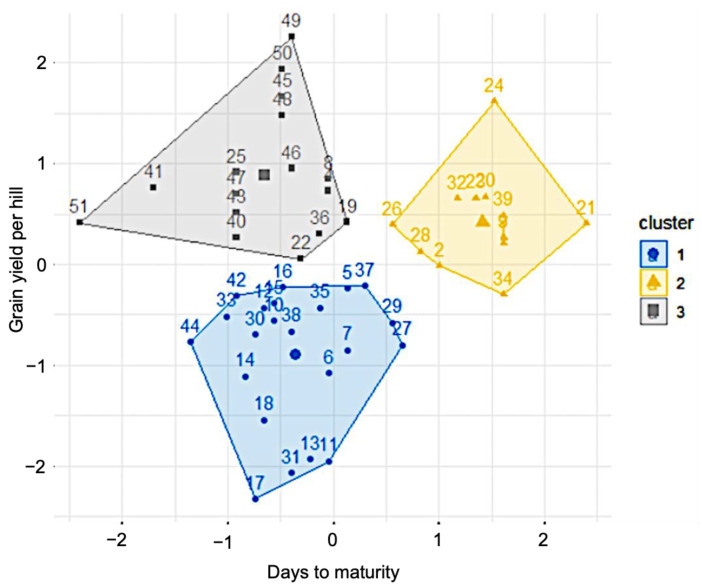
K-means cluster analysis of 50 F_5_ Aus rice accessions and one check variety BINAdhan-19 based on their grain yield per hill and days to maturity. The name of each genotype against its genotypic code is provided in Supplementary Table S2.

In order to further validate the genotype–trait association, we conducted a K-means clustering with a special focus on the DM and GY traits across the 51 genotypes (Figure 3). K-means clustering separated the investigated genotypes into three major clusters, as shown in Figure 3. Cluster 1 comprised the genotypes with low GY and moderate DM, cluster 2 displayed genotypes with longer DM and relatively high GY, while cluster 3 comprised genotypes with shorter DM and relatively higher GY (Figure 3). Remarkably, BU-R-ACC-02, BU-R-ACC-08 and R2-36-3-1-1 rice accessions, namely 41, 47 and 25 in Figure 3, were consistently grouped together in cluster 3 by K-means clustering analysis similar to that shown by the heatmap hierarchical clustering (Figure 2). This corroborative alignment not only confirmed the superior attributes of these accessions but also reaffirmed their potential significance in the realm of rice breeding for the development of short-duration and/or high-yielding cultivars.

## 3. Discussion

Breeding for rice genotypes that demonstrate attributes of shorter DM paired with enhanced GY presents a feasible and promising option to feed the growing global population [20,21]. In this study, 10 agronomic traits, including DM, ET, FG, GY, PH, PL, SY TT, TW and UFG, were examined across 50 F_5_ Aus rice accessions and a short-duration check variety BINAdhan-19 under field conditions (Table 1 and Supplementary Table S1). The objective was to identify the best genotypes characterized by short growth duration and high yielding potential under field conditions. Analysis of various yield and yield-associated traits revealed extensive phenotypic and genotypic variations within the examined Aus rice genotypes (Table 1 and Table 2 and Supplementary Table S1). These observations suggest that the agronomic diversity among the rice genotypes could be attributed to the interplay of genetic and environmental factors. Therefore, we carried out a multivariate genetic approach to identify the contributions of genetic and/or environmental components to the observed phenotypic variability in the investigated Aus rice germplasm. Specifically, we determined *σ*^2^*e*, *σ*^2^*g*, *σ*^2^*p*, ECV, GCV, PCV and *H*^2^ to assess the extent of inherent variation [22,23]. In the genetic model, the higher *σ*^2^*g* values than the *σ*^2^*e* values indicated the substantial genetic effects in influencing phenotypic expression [24]. Likewise, the *H*^2^ values were categorized into three groups: low (<30%), medium (30 to 60%) and high (>60%), aligning with the degree of genetic contribution to the observed phenotypic traits [25]. Additionally, the classification of GCV included low (<10%), moderate (10 to 20%) and high (>20%) categories, corresponding to the varying levels of genetic effects on the observed phenotypic traits [26,27]. If the GCV surpasses ECV, the genetic factors play a more significant influence in determining phenotypic variations [24]. Our investigation indicated that the *σ*^2^*g* values for DM, PH, PL and TW were notably greater than their corresponding *σ*^2^*e* values, and their *H*^2^ values were >60%, implying that these traits are primarily controlled by genetic factors (Table 2). Moreover, FG, UFG, GY, ET, TT and SY exhibited a moderate *H*^2^ range, spanned from 39.34 to 58.63%, indicating that these traits are influenced by both genetic and environmental factors (Table 2). Several reports [28,29,30] also reported that traits, such as DM, PH, PL and TW with high *H*^2^ and FG, GY and TT with moderate *H*^2^, are influenced by genetic and environmental factors, respectively, in different rice populations.

The *r*-values may be classified into six groups, three for positive and three for negative correlations: (i) low (0 to 0.30), (ii) medium (0.30 to 0.70) and (iii) high (0.70 to 1) for positive linear correlations, while (iv) low (0 to −0.30), (v) medium (−0.30 to −0.70) and (vi) high (−0.70 to −1) for negative linear correlations [27]. Our phenotype-based correlation analysis indicated that DM had a low positive correlation with TT and SY, whereas such a relationship between DM and other agronomical traits was absent in genotype-based correlation analysis (Figure 1a,b). It is worth noting that many of the investigated Aus rice accessions had a moderate DM compared with the BINAdhan-19 check variety, which might be the reason behind the absence of correlation between DM and other agronomic traits in the genotype-based correlation analysis (Supplementary Table S2). With respect to yield, the GY trait exhibited a low positive correlation with TW, FG, PL, ET and TT according to the phenotype-based correlation analysis (Figure 1a). GY also showed a low to moderate positive correlation with TW, FG and PL traits following a genotype-based correlation analysis (Figure 1b). Hence, in both phenotype-based and genotype-based correlation analyses, TW, FG and PL displayed a low to moderate positive association with GY, implying their notable role in influencing yield within our Aus rice population. Low to moderate positive correlations were also observed for TW, ET, FG and PH when investigated with GY in rice, as reported by Pratap et al. [31]. Li et al. [32] also observed that total grain weight per panicle (the sum of FG and UFG) exhibited moderate positive correlations with the harvest index. Similar to our findings, Strivasava and Tripathi [33] demonstrated that the maximum GY in rice was linked to increased FG, PL and TW. Faysal et al. [34] also revealed a positive correlation between the maximum GY in rice and the increase in FG and PL. Thus, these TW, FG and PL traits can be considered indicative markers when selecting breeding materials aimed at producing high-yield rice varieties.

Previous studies reported that BINAdhan-19 is a standard short-duration Aus variety with a semi-dwarf phenotype [35,36]. In this study, the check variety BINAdhan-19 had the lowest DM, followed by the accessions BU-R-ACC-02, BU-R-ACC-05, R3-49-2-1-1, R2-36-3-1-1, BU-R-ACC-01, BU-R-ACC-03, BU-R-ACC-04, BU-R-ACC-08 and R1-29-6-1-1, when compared with other investigated rice accessions (Figure 2 and Supplementary Table S2). Therefore, our comparative study indicated that some of the investigated rice accessions had relatively short DM similar to BINAdhan-19, which could be selected for the development of short-duration rice varieties. Currently, rice breeders are paying close attention to developing short-duration, high-yielding genotypes to culminate in maximum yield from a given land within a single year [37]. In the present study, K-means and hierarchical cluster analyses demonstrated that BU-R-ACC-02, BU-R-ACC-08 and R2-36-3-1-1 accessions have relatively higher GY and shorter DM than other Aus rice accessions (Figure 3), indicating that these genotypes have the best combination in terms of DM and GY and can be candidate genotypes for short DM rice breeding. As this investigation was carried out only in the F_5_ generation, future research should be considered to verify their similar characteristics in succeeding generations.

## 4. Materials and Methods

### 4.1. Experimental Details and Materials

The field experiment was performed at the field of Bangabandhu Sheikh Mujibur Rahman Agricultural University between March and July 2021. The experimental site was situated at a latitude of 24°05′ north and a longitude of 90°16′ east, positioned 8.4 m above the mean sea level within the agroecological zone (AEZ) twenty-eight. The experimental site’s soil is a type of shallow red-brown terrace soil, which is locally called the Salna series. It possesses less-than-optimal physical and chemical attributes and is characterized by a predominantly heavy clay texture, acidity and a silt loam composition in the upper 50 cm layer. This soil type falls under the category of typic Paleudults as per the United States Department of Agriculture classification, specifically belonging to the Orchrept suborder within the Inceptisol order [38]. For the experimental purpose of upland crops, the topsoil (up to 15 cm depth) of the experimental field was blended with imported alluvial soil from the adjacent floodplain. The experimental site experiences three distinct climatic seasons typical of subtropical regions: summer, rainy and winter, corresponding to the rice cultivation periods of Aus, Aman and Boro, respectively. Throughout the experimental duration spanning from March to July 2021, comprehensive records (https://bsmrau.edu.bd/age/weather-data/; accessed on 25 August 2021) were maintained for the monthly average temperatures, humidity levels, rainfall patterns and evaporation rates at the study location (Table 3).

This study employed a selection of 50 F_5_ Aus rice accessions in conjunction with the suitable reference variety BINAdhan-19 (Supplementary Table S2). BINAdhan-19 is a well-recognized Aus rice cultivar widely adopted in Bangladesh, which was released by the Bangladesh Institute of Nuclear Agriculture. The 50 Aus rice accessions under investigation were derived from nine distinct parent plants of the Aus rice variety. These successive generations were developed through a hybridization process utilizing the complete diallel-crossing technique, incorporating the parental lines Dhalasaitta, Laksmilota, Kataktara, Narica-ABSS, BRRI dhan43, BRRI dhan55, BR7, Nipponbare and Parija. These parent lines were sourced from diverse local and foreign origins. The pedigree of succession generation is presented in Supplementary Table S3.

### 4.2. Design of the Experiment and Data Recording

The experiment was carried out in a randomized complete block design comprising three independent blocks; each block represented one replication. The two blocks were separated by one meter. For each genotype, there were 30 unique plants in each replication. The plant spacing was 20 cm × 15 cm throughout the experimental plot. Three rice seeds were sown per hill. To ensure the uniform germination of seeds, light irrigation was provided. In order to fertilize the plants, urea (as a source of nitrogen (N)), triple super phosphate (as a source of phosphorus (P)) and muriate of potash (as a source of potassium (K)) were used. NPK fertilizers were applied at the rate of 175, 80 and 110 Kg, respectively, per ha. P and K fertilizers were applied during the final land preparation, whereas N fertilizer was applied in three splits. In each split, 58.34 Kg of N fertilizer was applied for each ha. The first split of N fertilizer was added to the soil during the final land preparation. The second and third splits of N fertilizer were administered at 35 and 55 days after sowing, respectively. Each plot underwent uniform intercultural activities, such as irrigation, weeding, drainage and plant protection measures to guarantee that the rice crop grew normally.

Plants were harvested when 85% of the grains of the panicles turned a golden yellow color. Three biological replicates (*n* = 3; 5 plants per replicate) were used to collect data on growth, yield and yield-related traits. The studied DM trait was measured from the sowing to harvesting day. At harvest, PH was determined from the soil surface to the highest part of the plant; TT was counted as the number of tillers per hill when the tillers had at least one leaf; ET was counted as the number of panicles bearing tillers per hill; PL was determined from the bottom of the peduncle to the topmost of the panicle; FG and UFG were counted when spikelets had endosperm and without endosperm, respectively. TW, GY and SY were all measured in grams and corrected to 14% moisture content (MC) using formula 1 as follows:

(1) $$TW/GY/SY\;at\;14\%\;MC=\frac{100-SMC}{100-DMC}\times SFW$$

where SMC is the moisture content of the sample, DMC is the desired moisture content and SFW is the sample fresh weight.

To quantify genetic diversity among genotypes and evaluate the influence of both genetic and environmental factors were computed as follows:

Environmental variance (σ²e), genotypic variance (*σ*^2^*g*) and phenotypic variance (*σ*^2^*p*) were estimated by formulae 2, 3 and 4, respectively, according to Faysal et al. [34]

(2) $$\sigma^{2}e=EMS$$

where EMS is the error mean square.

(3) $$\sigma^{2}g=\frac{GMS-EMS}{R}$$

where GMS is the genotypic mean square, EMS is the error mean square, and R is the number of replications.

(4) $$\sigma^{2}p=\sigma^{2}g+\sigma^{2}e$$

The environmental coefficient of variation (ECV) percentage was calculated by formula 5 according to Azimi et al. [39]

(5) $$ECV\left(\%\right)=\frac{\sigma^{2}e}{\bar{X}}\times 100$$

where $\bar{X}$ represents the population mean.

The genotypic coefficient of variation (GCV) percentage and phenotypic coefficient of variation (PCV) percentage were estimated by formulae 6 and 7, respectively, according to Faysal et al. [34]

(6) $$GCV\left(\%\right)=\frac{\sigma^{2}g}{\bar{X}}\times 100$$

(7) $$PCV\left(\%\right)=\frac{\sigma^{2}p}{\bar{X}}\times 100$$

Broad-sense heritability (*H*^2^) percentage was estimated by formula 8 according to Burton [40]

(8) $$H^{2}\left(\%\right)=\frac{\sigma^{2}g}{\sigma^{2}p}\times 100$$

### 4.3. Statistical Analysis

The one-way ANOVA, descriptive statistics and Fisher’s LSD test of the investigated parameters were conducted by the ‘doebioresearch’ package in R 3.2.0 (https://www.r-project.org/ accessed on 17 February 2023). The genotype-based and phenotype-based correlation analyses were carried out using the ‘variability’ and ‘ggcorrplot’ packages in R. K-means clustering and heatmap hierarchical cluster analysis were carried out using the ‘factoextra’ and ‘cluster’ packages in R 3.2.0, respectively.

## 5. Conclusions

Significant variations were evident across all 10 investigated agronomical traits within the cohort of 50 F_5_ Aus rice genotypes alongside the BINAdhan-19 check variety. Among these attributes, FG, PL and TW demonstrated substantial correlations with GY as deduced from both phenotype-based and genotype-based correlation analyses. This indicates the viability of utilizing these traits as discerning markers for germplasm selection. Certain genotypes exhibited shorter DM characteristics similar to the BINAdhan-19 check variety. Notably, the Aus rice accessions BU-R-ACC-02, BU-R-ACC-08 and R2-36-3-1-1 demonstrated a remarkable combination of shorter DM and heightened GY within optimal field conditions. In the future, these rice accessions need to be examined under diverse agronomic management practices, thereby substantiating their performance and adaptability across varied environmental contexts. Hence, these identified accessions warrant consideration as prospective germplasm candidates for inclusion within rice breeding programs aimed at the development of short-growth cycle, high-yielding rice varieties endowed with favorable agronomic attributes.

## Figures and Tables

**Table 1:** Comparative descriptive statistics of 10 agronomical traits in 50 F_5_ Aus rice accessions and check variety BINAdhan-19.

Source of Variation	Replication		Genotype	
df (*n* − 1)	2		50	
Agronomical Traits	Mean	MD	LSD_0.05_	CV (%)
Days to maturity (DM)	106.17 ± 3.85	9.35	1.20	0.70
Plant height (PH)	114.61 ± 12.83	25.75	8.12	4.38
Number of total tillers per hill (TT)	13.38 ± 2.65	5.19	3.17	14.63
Number of effective tillers per hill (ET)	11.72 ± 2.48	4.00	3.08	16.23
Panicle length (PL)	25.40 ± 2.25	3.59	2.16	5.25
Number of filled grains per panicle (FG)	122.05 ± 35.86	64.39	37.31	18.87
Number of unfilled grains per panicle (UFG)	28.73 ± 19.46	38.67	20.40	43.84
1000-grain weight (TW)	23.94 ± 3.47	3.39	1.95	5.04
Grain yield per hill (GY)	23.84 ± 5.57	7.67	6.37	6.50
Straw yield per hill (SY)	26.06 ± 5.60	10.29	6.93	6.43

Values (means ± standard deviation) were derived from 51 rice genotypes using three biological replicates (*n* = 3; 5 plants per replicate). CV, coefficient of variation; df, degree of freedom; LSD, least significant difference; MD, mean difference. The df (*n* − 1) formula indicates sample size minus one parameter tested.

**Table 2:** Estimates of environmental variance (*σ*^2^*e*), genotypic variance (*σ*^2^*g*), phenotypic variance (*σ*^2^*p*), environmental coefficient of variation (ECV) percentages, genotypic coefficient of variation (GCV) percentages, phenotypic coefficient of variation (PCV) percentages and broad-sense heritability (*H*^2^) percentages of 50 F_5_ Aus rice accessions and BINAdhan-19 check variety for the investigated traits.

Sources of Variation	DM	PH	TT	ET	PL	FG	UFG	TW	GY	SY
Mean	106.17	114.61	13.38	11.72	25.40	122.05	28.73	23.94	23.84	26.06
*σ* ^2^ *e*	0.55	25.16	3.83	3.62	1.78	530.48	158.63	1.46	15.48	18.34
*σ* ^2^ *g*	14.41	140.99	3.06	2.35	3.35	749.17	224.77	10.75	15.64	13.02
*σ* ^2^ *p*	14.97	166.16	6.89	5.97	5.13	1279.65	383.40	12.20	31.12	31.35
ECV (%)	0.70	4.38	14.63	16.23	5.25	18.87	43.84	5.04	16.50	16.43
GCV (%)	3.58	10.36	13.08	13.07	7.21	22.43	52.19	13.69	16.59	13.85
PCV (%)	3.64	11.25	19.62	20.84	8.92	29.31	68.16	14.59	23.40	21.49
*H*^2^ (%)	96.31	84.86	44.41	39.34	65.37	58.54	58.63	88.07	50.26	41.52

DM, days to maturity; ET, number of effective tillers per hill; FG, number of filled grains per panicle; GY, grain yield per hill; PH, plant height; PL, panicle length; SY, straw yield per hill; TT, number of total tillers per hill; TW, 1000-grain weight; UFG, number of unfilled grains per panicle.

**Table 3:** Metrological conditions prevailed during the Aus season 2021 at Bangabandhu Sheikh Mujibur Rahman Agricultural University, Gazipur, Bangladesh.

Month	Air Temperature (°C)			Humidity (%)	Rainfall (mm)	Evaporation (mm)
	Max.	Min.	Ave.			
March	34.39	21.23	27.81	81.90	4.30	121.18
April	36.05	24.13	30.09	80.57	62.01	160.30
May	35.21	25.45	30.33	82.00	162.01	152.19
June	32.93	26.72	29.83	87.33	490.58	151.56
July	32.82	26.98	29.90	86.45	398.21	130.91
Mean	34.28	24.90	29.59	83.65	223.42	143.23

## Data Availability

All data generated or analyzed during this study are included in this published article.

## Supplementary Materials

The following supporting information can be downloaded at: https://www.mdpi.com/article/10.3390/plants12203601/s1, Table S1: Descriptive statistics and analysis of variance of investigated agronomical traits in 50 F_5_ Aus rice accessions and check variety BINAdhan-19; Table S2: Mean values of investigated agronomical traits in 50 F_5_ Aus rice accessions and check variety BINAdhan-19; Table S3: Pedigree of 50 Aus rice accessions in F_5_ generation.
