# Supplementary material for: Exploring the Phenotypic and Genetic Variabilities in Yield and Yield-Related Traits of the Diallel-Crossed F5 Population of Aus Rice
Source: Plants (Basel). 2023 Oct 17;12(20):3601. doi: 10.3390/plants12203601 (PMC10610382; doi:10.3390/plants12203601)
Supplement: Supplementary file 1 [file plants-12-03601-s001.zip › plants-2491578-supplementary.pdf]

Table S1. Descriptive statistics and analysis of variance (ANOVA) of investigated agronomical traits in 50 F_5 Aus rice accessions and check variety BINAdhan-19.

**Agronomical traits**	**Maximum**	**Minimum**	**Skewness**	**Kurtosis**	**SS**	**MSS**	***F*-value**	***p*-value**
Days to maturity (DM)	116.00	96.00	0.38	-0.19	2189.58	43.79	79.32	<2e-16
Plant height (PH)	150.20	85.88	0.57	0.08	22407.20	448.14	17.81	<2e-16
Number of total tillers per hill (TT)	21.20	7.00	0.52	0.31	650.48	13.01	3.39	9.99e-08
Number of effective tillers per hill (ET)	19.40	7.00	0.59	0.41	533.55	10.67	2.94	2.21e-06
Panicle length (PL)	35.63	20.40	0.97	2.43	591.78	11.84	6.66	5.53e-16
Number of filled grains per panicle (FG)	267.33	76.00	1.49	2.49	138899.80	2777.99	5.23	1.08e-12
Number of unfilled grains per panicle (UFG)	115.00	3.00	2.00	4.39	41647.12	832.94	5.25	9.93e-13
1000-grain weight (TW)	31.28	13.03	-0.37	-0.02	1684.58	33.70	23.15	<2e-16
Grain yield per hill (GY)	39.35	11.03	0.21	0.20	3120.38	62.41	4.03	1.54e-09
Straw yield per hill (SY)	45.04	16.06	0.83	0.52	2869.39	57.39	3.12	6.18e-07

Trait values represent the means of three independent biological replicates (n = 3; 5 plants per replicate). Skewness and kurtosis were obtained from descriptive statistics. SS, MSS, F-value and p-value were derived from ANOVA. MSS, mean sum of squares; SS, sum of square.

Table S2. Mean values of investigated agronomical traits in 50 F_5 Aus rice accessions and check variety BINAdhan-19.

**Genotypes**	**Code**	**DM**	**PH**	**TT**	**ET**	**PL**	**FG**	**UFG**	**TW**	**GY**	**SY**
R1-6-2-1-1	1	112.33^{b}	107.52^{o-t}	12.27^{f-p}	10.47^{h-p}	23.46^{n-t}	90.67^{h-j}	12.56^{kl}	28.66^{a-d}	24.78^{e-m}	27.36^{d-l}
R1-48-3-1-1	2	110.00^{de}	107.12^{o-t}	12.60^{f-p}	11.27^{e-p}	27.54^{b-f}	123.89^{e-i}	44.56^{c-g}	23.95^{l-r}	23.79^{f-m}	25.40^{e-n}
R1-9-10-1-1	3	112.33^{b}	103.27^{s-v}	12.73^{f-p}	10.73^{g-p}	23.37^{o-t}	161.22^{b-d}	18.44^{i-l}	20.27^{w-y}	25.08^{d-m}	23.20^{i-o}
R1-44-1-1-1	4	106.00^{ij}	110.15^{n-s}	14.80^{c-i}	12.73^{b-k}	26.00^{e-l}	121.33^{e-i}	17.00^{j-l}	23.71^{l-s}	27.18^{b-i}	24.90^{f-n}
R1-52-1-1-1	5	106.67^{hi}	105.14^{r-u}	15.27^{b-g}	12.47^{c-m}	25.39^{f-p}	100.67^{h-j}	19.89^{h-l}	22.87^{n-v}	22.77^{f-n}	22.46^{j-o}
R1-47-3-1-1	6	106.00^{ij}	121.91^{c-i}	13.00^{e-p}	11.13^{e-p}	23.37^{o-t}	90.89^{h-j}	30.56^{d-k}	24.07^{k-r}	18.94^{l-q}	20.46^{l-o}
R1-17-1-1-1	7	106.67^{hi}	111.43^{l-r}	14.20^{d-l}	12.20^{d-n}	25.27^{g-q}	108.56^{h-j}	19.11^{h-l}	21.23^{v-x}	19.96^{k-p}	22.00^{k-o}
R1-13-1-1-1	8	106.00^{ij}	127.77^{c-e}	12.20^{g-p}	10.47^{h-p}	25.96^{e-l}	161.89^{b-d}	8.67^{l}	26.72^{d-i}	27.70^{b-g}	28.49^{b-k}
R1-54-4-1-1	9	112.33^{b}	126.79^{c-f}	12.60^{f-p}	11.27^{e-p}	26.96^{b-h}	104.67^{h-j}	26.33^{f-l}	28.29^{b-e}	25.04^{d-m}	25.88^{e-n}
R1-49-7-1-1	10	104.00^{m-p}	105.53^{q-u}	11.17^{k-p}	9.33^{n-p}	26.18^{e-l}	114.11^{g-j}	23.11^{h-l}	22.11^{r-w}	21.28^{h-o}	20.25^{m-o}
R1-56-2-1-1	11	106.00^{ij}	104.61^{r-v}	13.95^{e-p}	12.03^{e-o}	26.33^{d-j}	105.56^{h-j}	16.22^{j-l}	20.06^{xy}	14.94^{o-q}	19.79^{no}
R1-13-2-1-1	12	103.67^{n-q}	119.40^{f-l}	10.33^{op}	9.67^{k-p}	25.48^{f-o}	152.11^{c-f}	16.44^{j-l}	23.86^{l-r}	21.89^{f-n}	26.66^{e-n}
R1-24-3-1-1	13	105.33^{j-l}	94.07^{wx}	12.47^{f-p}	11.47^{e-p}	21.72^{t}	198.44^{ab}	94.44^{a}	15.38	15.03^{o-q}	16.52^{o}
R1-29-6-1-1	14	103.00^{p-r}	129.73^{bc}	10.93^{m-p}	9.27^{n-p}	28.40^{b-d}	118.33^{f-j}	23.67^{h-l}	29.16^{a-c}	18.79^{m-q}	30.93^{b-g}
R1-43-1-1-1	15	104.00^{m-p}	105.44^{r-u}	15.40^{b-f}	14.07^{a-f}	25.10^{h-q}	110.22^{h-j}	31.22^{d-k}	22.25^{q-v}	22.09^{f-n}	26.19^{e-n}
R1-50-1-1-1	16	104.33^{l-o}	105.62^{q-u}	14.80^{c-i}	12.60^{b-l}	26.53^{c-i}	113.11^{g-j}	24.00^{h-l}	22.86^{n-v}	22.83^{f-n}	24.37^{f-n}
R1-19-4-1-1	17	103.33^{o-r}	117.68^{g-n}	11.08^{l-p}	9.67^{k-p}	22.73st	149.11^{c-g}	49.89^{c-e}	15.85	13.21^{q}	21.16^{l-o}
R2-30-9-1-1	18	103.67^{n-q}	137.33^{ab}	12.40^{f-p}	10.73^{g-p}	24.28^{j-s}	97.22^{h-j}	29.89^{e-k}	23.51^{m-t}	16.81^{n-q}	30.86^{b-g}
R2-56-7-1-1	19	106.67^{hi}	107.17^{o-t}	18.08^{ab}	15.22^{a-d}	25.18^{h-q}	93.89^{h-j}	13.56^{kl}	18.87^{yz}	25.76^{c-k}	25.63^{e-n}
R2-2-10-1-1	20	111.67^{bc}	118.48^{g-m}	17.07^{a-d}	14.00^{a-f}	25.76^{e-m}	109.89^{h-j}	19.78^{h-l}	24.08^{k-q}	26.91^{b-j}	33.72^{a-d}
R2-30-4-1-1	21	115.33^{a}	107.73^{o-t}	15.93^{a-e}	14.20^{a-e}	23.19^{q-t}	180.00^{a-c}	60.67^{bc}	18.09^{z}	25.70^{c-k}	34.87^{a-c}
R2-15-7-1-1	22	105.00^{j-m}	104.40^{r-v}	14.53^{c-j}	13.80^{a-g}	25.40^{f-p}	107.33^{h-j}	16.67^{j-l}	23.23^{m-u}	24.09^{f-m}	21.82^{k-o}
R2-6-14-1-1	23	111.33^{bc}	141.87^{a}	14.58^{c-j}	13.18^{b-i}	28.59^{bc}	117.67^{f-j}	31.89^{d-k}	26.90^{d-i}	26.85^{b-j}	35.19^{ab}
R2-26-6-1-1	24	112.00^{b}	119.66^{e-k}	13.20^{e-p}	12.53^{b-m}	24.56^{i-s}	118.00^{f-j}	12.89^{kl}	25.99^{f-k}	31.22^{a-d}	28.62^{b-k}
R2-36-3-1-1	25	102.67^{qr}	129.34^{b-d}	11.73^{i-p}	10.53^{h-p}	24.18^{j-s}	168.56^{bc}	75.78^{ab}	20.17^{w-y}	28.02^{a-f}	26.99^{d-m}
R2-29-1-1-1	26	108.33^{fg}	96.86^{v-x}	13.73^{e-n}	12.27^{d-n}	24.40^{i-s}	167.44^{bc}	50.78^{cd}	20.12^{xy}	25.65^{c-k}	21.97^{k-o}
R2-56-6-1-1	27	108.67^{f}	98.55^{u-w}	18.80^{a}	16.47^{a}	23.28^{p-t}	88.00^{ij}	14.44^{kl}	21.60^{t-x}	20.19^{k-p}	22.92^{i-o}
R2-43-3-1-1	28	109.33^{ef}	108.13^{o-t}	13.53^{e-n}	12.27^{d-n}	25.60^{e-n}	112.78^{g-j}	16.22^{j-l}	22.65^{o-v}	24.42^{e-m}	22.47^{j-o}
R2-31-1-1-1	29	108.33^{fg}	139.13^{a}	12.00^{h-p}	10.00^{j-p}	27.39^{b-g}	107.00^{h-j}	24.33^{g-l}	21.604^{t-x}	21.20^{h-o}	28.14^{c-k}
R2-54-3-1-1	30	103.33^{o-r}	140.13^{a}	10.60^{n-p}	9.67^{k-p}	25.06^{h-r}	104.00^{h-j}	25.67^{g-l}	27.25^{c-g}	20.66^{j-p}	25.73^{e-n}
R2-49-3-1-1	31	104.67^{k-n}	106.15^{p-u}	13.00^{e-p}	11.00^{f-p}	22.93^{r-t}	91.11^{h-j}	17.89^{i-l}	25.04^{i-m}	14.42^{pq}	22.40^{j-o}

R3-10-5-1-1	32	110.67 ^{cd}	141.27 ^{a}	10.13 ^{p}	9.33 $^{n-p}$	33.27 ^{a}	121.44 $^{e-i}$	46.33 $^{c-f}$	29.67 ^{ab}	26.83 $^{b-j}$	30.56 $^{b-h}$
R3-49-2-1-1	33	102.33 ^{r}	125.40 $^{c-g}$	13.53 $^{e-n}$	12.07 $^{e-o}$	24.13 $^{k-s}$	118.67 $^{f-j}$	37.56 $^{d-i}$	24.50 $^{k-o}$	21.50 $^{g-n}$	23.65 $^{h-n}$
R3-26-6-1-1	34	112.33 ^{b}	115.22 $^{h-o}$	13.33 $^{e-o}$	12.00 $^{e-o}$	23.17 $^{q-t}$	83.22 ^{j}	27.67 $^{f-l}$	25.93 $^{g-k}$	22.48 $^{f-n}$	29.21 $^{b-j}$
R3-46-8-1-1	35	105.67 $^{i-k}$	121.87 $^{c-i}$	14.40 $^{c-j}$	12.20 $^{d-n}$	23.71 $^{m-t}$	111.33 $^{h-j}$	16.33 $^{j-l}$	21.78 $^{s-x}$	21.89 $^{f-n}$	25.67 $^{e-n}$
R3-45-5-1-1	36	105.67 $^{i-k}$	111.31 $^{l-s}$	14.80 $^{c-i}$	13.47 $^{a-h}$	25.07 $^{h-r}$	107.33 $^{h-j}$	25.00 $^{g-l}$	24.25 $^{k-p}$	25.22 $^{c-l}$	24.14 $^{g-n}$
R3-15-3-1-1	37	107.33 ^{gh}	108.50 $^{o-t}$	14.60 $^{c-j}$	12.53 $^{b-m}$	26.10 $^{e-l}$	116.56 $^{f-j}$	25.67 $^{g-l}$	22.39 $^{p-v}$	22.88 $^{f-n}$	22.61 $^{i-o}$
R3-49-4-1-1	38	104.67 $^{k-n}$	117.59 $^{g-n}$	14.33 $^{c-k}$	12.80 $^{b-j}$	23.62 $^{m-t}$	98.67 $^{h-j}$	28.44 $^{f-l}$	23.27 $^{m-u}$	20.82 $^{i-o}$	22.87 $^{i-o}$
R3-20-6-1-1	39	112.33 ^{b}	122.09 $^{c-h}$	13.93 $^{d-m}$	11.33 $^{e-p}$	23.78 $^{m-t}$	94.33 $^{h-j}$	21.89 $^{h-l}$	27.40 $^{c-g}$	26.07 $^{c-k}$	31.27 $^{b-f}$
BU-R-ACC-01	40	102.67 ^{qr}	115.05 $^{h-o}$	17.47 $^{a-c}$	15.60 ^{ab}	27.67 $^{b-e}$	103.67 $^{h-j}$	35.44 $^{d-j}$	27.11 $^{d-g}$	25.04 $^{d-m}$	28.22 $^{c-k}$
BU-R-ACC-02	41	99.67 ^{t}	121.55 $^{d-j}$	15.13 $^{b-h}$	13.13 $^{b-i}$	24.07 $^{l-s}$	91.11 $^{h-j}$	23.78 $^{h-l}$	30.39 ^{a}	27.33 $^{b-h}$	32.21 $^{a-e}$
BU-R-ACC-03	42	102.67 ^{qr}	113.57 $^{j-q}$	11.20 $^{k-p}$	9.60 $^{l-p}$	26.08 $^{e-l}$	125.44 $^{d-h}$	27.67 $^{f-l}$	26.80 $^{d-i}$	22.43 $^{f-n}$	38.21 ^{a}
BU-R-ACC-04	43	102.67 ^{qr}	121.01 $^{e-j}$	10.67 $^{n-p}$	9.33 $^{n-p}$	25.53 $^{e-n}$	103.11 $^{h-j}$	28.67 $^{f-l}$	27.04 $^{d-h}$	26.19 $^{c-k}$	29.44 $^{b-i}$
BU-R-ACC-05	44	101.00 ^{s}	111.59 $^{l-r}$	12.00 $^{h-p}$	10.60 $^{h-p}$	24.72 $^{i-s}$	125.44 $^{d-h}$	18.11 $^{i-l}$	26.98 $^{d-i}$	20.33 $^{k-p}$	25.97 $^{e-n}$
BU-R-ACC-06	45	104.33 $^{l-o}$	113.79 $^{i-p}$	16.93 $^{a-d}$	15.40 $^{a-c}$	24.27 $^{j-s}$	96.78 $^{h-j}$	26.89 $^{f-l}$	21.40 $^{u-x}$	31.46 $^{a-c}$	27.10 $^{d-m}$
BU-R-ACC-07	46	104.67 $^{k-n}$	108.15 $^{o-t}$	11.53 $^{j-p}$	9.47 $^{m-p}$	28.87 ^{b}	178.22 ^{bc}	32.44 $^{d-k}$	26.54 $^{e-j}$	28.19 $^{a-f}$	26.09 $^{e-n}$
BU-R-ACC-08	47	102.67 ^{qr}	108.79 $^{o-s}$	12.00 $^{h-p}$	9.07 ^{op}	25.38 $^{g-p}$	153.56 $^{c-f}$	62.89 ^{bc}	25.15 $^{h-m}$	27.02 $^{b-j}$	25.66 $^{e-n}$
BU-R-ACC-09	48	104.33 $^{l-o}$	110.85 $^{m-s}$	14.53 $^{c-j}$	13.07 $^{b-j}$	26.27 $^{d-k}$	127.78 $^{d-h}$	39.11 $^{d-h}$	21.48 $^{u-x}$	30.58 $^{a-e}$	25.22 $^{f-n}$
BU-R-ACC-10	49	104.67 $^{k-n}$	109.35 $^{o-s}$	11.87 $^{i-p}$	10.13 $^{i-p}$	28.59 ^{bc}	158.00 $^{c-e}$	16.22 $^{j-l}$	27.90 $^{b-f}$	34.14 ^{a}	29.41 $^{b-i}$
BU-R-ACC-11	50	104.33 $^{l-o}$	100.45 $^{t-w}$	10.07 ^{p}	8.40 ^{p}	26.30 $^{d-j}$	216.11 ^{a}	16.44 $^{j-l}$	24.77 $^{j-n}$	32.67 ^{ab}	22.08 $^{k-o}$
BINA dhan-19	51	97.00 ^{u}	89.40 ^{x}	11.67 $^{i-p}$	11.67 $^{e-o}$	25.20 $^{h-q}$	106.00 $^{h-j}$	22.00 $^{h-l}$	25.62 $^{g-l}$	25.73 $^{c-k}$	21.87 $^{k-o}$
LSD$_{0.05}$		1.20	8.20	3.20	3.10	2.20	37.30	20.40	1.90	6.40	6.90
CV (%)		0.70	4.38	14.60	16.20	5.30	18.80	43.80	5.10	16.50	16.40

Trait values were derived from three independent biological replicates (*n* = 3; 5 plants per replicate). Different alphabetic letters in the column indicates significant differences among genotypes according to a Fisher's least significant difference (LSD$_{0.05}$) test. CV, coefficient of variation; DM, days to maturity; ET, number of effective tillers per hill; FG, number of filled grains per panicle; GY, grain yield per hill; PH, plant height; PL, panicle length; SY, straw yield per hill; TT, number of total tillers per hill; TW, 1000-grain weight; UFG, number of unfilled grains per panicle.

Table S3. Pedigree of 50 Aus rice accessions in F_5 generation.

**Name of the parents**		**Male parent (♂)**								
		Dhalasaitta (SD+MY)	Laksmilota (SD+MY)	Kataktara (SD+MY)	Narica-ABSS (DT+MY)	BRRI dhan43 (LD+HY)	BRRI dhan55 (LD+HY)	BR7 (LD+HY)	Nipponbare (DT+MY)	Parija (SD+LY)
**Female parent (♀)**	Dhalasaitta (SD+MY)	–	R_1-6-2-1-1	R_1-48-3-1-1	R_2-54-3-1-1	R_2-36-3-1-1	R_1-17-1-1-1	R_1-43-1-1-1	–	–
	Laksmilota (SD+MY)	R_2-29-1-1-1	–	–	R_1-44-1-1-1	R_2-30-9-1-1	R_3-49-2-1-1	R_1-52-1-1-1	–	R_1-47-3-1-1
	Kataktara SD+MY)	R_1-9-10-1-1	–	–	–	R_2-56-7-1-1	R_1-50-1-1-1	R_1-13-1-1-1	–	R_2-6-14-1-1
	Narica-ABSS (DT+MY)	R_2-15-7-1-1	R_2-56-6-1-1	R_1-29-6-1-1	–	R_2-2-10-1-1	BU-R-ACC-05	BU-R-ACC-06	–	–
	BRRI dhan43 (LD+HY)	R_3-15-3-1-1	R_1-54-4-1-1	-	BU-R-ACC-01	–	BU-R-ACC-03	R_1-24-3-1-1	–	BU-R-ACC-11
	BRRI dhan55 (LD+HY)	R_1-49-7-1-1	R_2-30-4-1-1	BU-R-ACC-08	BU-R-ACC-09	R_3-46-8-1-1	–	BU-R-ACC-02	–	BU-R-ACC-10
	BR7 (LD+HY)	–	R_3-20-6-1-1	R_2-49-3-1-1	BU-R-ACC-04	R_1-56-2-1-1	R_3-45-5-1-1	–	R_3-49-4-1-1	–
	Nipponbare (DT+MY)	–	R_1-19-4-1-1	–	R_2-26-6-1-1	BU-R-ACC-07	R_3-10-5-1-1	–	–	–
	Parija (EM+LY)	–	R_3-26-6-1-1	R_2-43-3-1-1	–	–	R_1-13-2-1-1	–	R_2-31-1-1-1	–

The accessions were developed through hybridization between the male and female parents. "–" indicates the empty accession. The succeeding generations were developed through hybridization between the parents of Dhalasaitta, Laksmilota, Kataktara, Narica-ABSS, BRRI dhan43, BRRI dhan55, BR7, Nipponbare, and Parija obtained from various local and exotic sources. DT, HY, LD, LY, MY and SD indicate drought-tolerant, high-yielding, long-duration, low-yielding, medium-yielding and short-duration characteristics, respectively.
